# The effect of spaceflight and microgravity on the human brain

**DOI:** 10.1007/s00415-017-8427-x

**Published:** 2017-03-07

**Authors:** Angelique Van Ombergen, Athena Demertzi, Elena Tomilovskaya, Ben Jeurissen, Jan Sijbers, Inessa B. Kozlovskaya, Paul M. Parizel, Paul H. Van de Heyning, Stefan Sunaert, Steven Laureys, Floris L. Wuyts

**Affiliations:** 1 Antwerp University Research Centre for Equilibrium and Aerospace (AUREA), University of Antwerp, Antwerp, Belgium; 2 Faculty of Medicine and Health Sciences, University of Antwerp, Antwerp, Belgium; 3 Faculty of Sciences, Department of Biomedical Physics, University of Antwerp, Antwerp, Belgium; 4 Coma Science Group, GIGA-Research & Neurology Department, University and University Hospital of Liège, Liège, Belgium; 5 Brain and Spine Institute-Institut du Cerveau et de la Moelle Épinière (ICM), Hôpital-Pitié-Salpêtrière, Paris, France; 6 SSC RF-Institute of Biomedical Problems, Russian Academy of Sciences, Moscow, Russia; 7 Imec/Vision Lab, Department of Physics, University of Antwerp, Antwerp, Belgium; 8 Department of Radiology, Antwerp University Hospital & University of Antwerp, Antwerp, Belgium; 9 Department of Imaging & Pathology, Translational MRI, KU Leuven-University of Leuven, Louvain, Belgium; 10 Antwerp University Research Centre for Equilibrium and Aerospace (AUREA), Department of Biomedical Physics, University of Antwerp, Groenenborgerlaan 171, 2020 Antwerpen, Belgium

**Keywords:** Human spaceflight, Microgravity, Brain, Central nervous system, Dry immersion, Bed rest, Parabolic flight, MRI, EEG, Neuroplasticity

## Abstract

Microgravity, confinement, isolation, and immobilization are just some of the features astronauts have to cope with during space missions. Consequently, long-duration space travel can have detrimental effects on human physiology. Although research has focused on the cardiovascular and musculoskeletal system in particular, the exact impact of spaceflight on the human central nervous system remains to be determined. Previous studies have reported psychological problems, cephalic fluid shifts, neurovestibular problems, and cognitive alterations, but there is paucity in the knowledge of the underlying neural substrates. Previous space analogue studies and preliminary spaceflight studies have shown an involvement of the cerebellum, cortical sensorimotor, and somatosensory areas and the vestibular pathways. Extending this knowledge is crucial, especially in view of long-duration interplanetary missions (e.g., Mars missions) and space tourism. In addition, the acquired insight could be relevant for vestibular patients, patients with neurodegenerative disorders, as well as the elderly population, coping with multisensory deficit syndromes, immobilization, and inactivity.

## Introduction

Half a century of manned spaceflight has taught us that space travel can have detrimental effects on human physiology. Due to the increasing duration of space missions from a few days up to several months and even a year, these detrimental effects have become clearer. Examples are visual impairment intracranial pressure (VIIP) syndrome, bone density loss, muscle atrophy, and neurovestibular problems.

It is likely that spaceflight, and microgravity in particular, will induce alterations in (electro)cortical function and structure for a number of reasons. One of them is the fact that the otoliths that constitute the linear acceleration detectors of the vestibular system are abruptly deprived of the sense of gravity. This hampered peripheral input could in turn have an effect on the vestibular nuclei, as well as on the cortical projections where integration of the different sensory inputs takes place, such as the parieto-insular region, the thalamus, and the temporoparietal region (e.g., [1] for a review on animal and human data). Furthermore, the recurrent observations of problems with movement-timing and motor coordination in returning space crew [2] suggest plausible alterations of structure and function of the cerebellum, responsible for coordination and fine motor control [3].

Currently, there is paucity in the knowledge on the exact effect of microgravity on the human brain. However, with an eye towards interplanetary missions and space tourism (introducing non-experienced and non-trained individuals to microgravity), it is important to get a better insight into the neural changes taking place. This will ensure crew safety and health by allowing the development of new and the optimization of current countermeasures. Since space studies are limited by several logistic, financial, and practical restrictions, ground-based analogues have been developed to overcome some of these problems. We will here give a concise overview of the current knowledge of the effect of microgravity on the brain, based on actual spaceflight as well as parabolic flight studies assessed with electroencephalography and magnetic resonance imaging, complemented with results from dry immersion and head-down bed rest space analogues.

## Space analogues

Dry immersion involves immersing the subject in thermo-neutral water while being covered in an elastic waterproof fabric to keep the subject dry. By doing so, there is no direct contact with water. Immersion is an adequate spaceflight alternative, since it mimics several spaceflight features, such as ‘supportlessness’ (i.e., lack of a supporting structure against the body), centralization of bodily fluids, confinement, immobilization, and hypokinesia [4].

Head-down bed rest (HDBR) is the most implemented space analogue. During HDBR, a subject lies in a bed that is inclined with the head down (−6° in most cases) typically for a period of 1 week, 1 month, or even longer. This HDBR causes a cephalic fluid shift and the absolute restriction to the bed replicates immobilization, isolation, and monotony of activities. However, the gravitational and vestibular input remains [5].

**Fig. 1:**
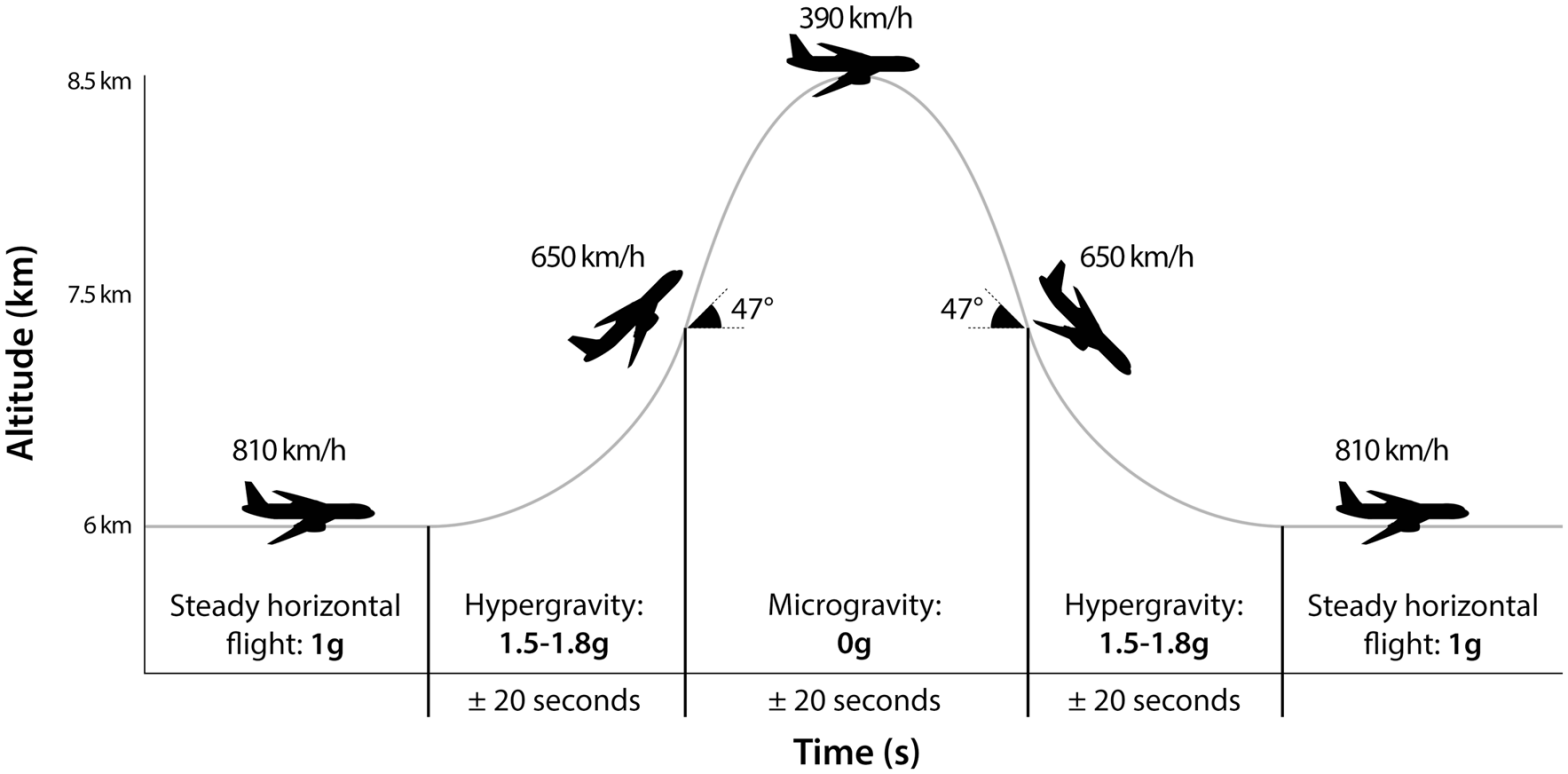
Typical flight trajectory of a parabolic flight for 0 g parabola’s

During a parabolic flight (PF), a specific flight trajectory is carried out by an airplane, such that normo-, hyper-, and microgravity phases are alternatingly experienced by the subjects on board of the aircraft (Fig. 1) [6]. In addition, this trajectory can be modified to acquire parabolas of Martian gravity (0.38 g) and lunar gravity (0.16 g). The typical duration of microgravity is of the order of 20 s, but the aircraft usually flies 15–30 parabolas during one mission. A PF is the only ground-based method allowing life science experiments in actual microgravity [6].

## Electroencephalography (EEG) studies

EEG has been commonly used to investigate electrocortical activity during microgravity. Due to its portability, EEG allows for easy implementation in an extreme environment such as space [7]. During EEG, electrical activity of the brain is monitored and measured by placing multiple electrodes along the scalp. EEG has a good temporal resolution, but a low(er) spatial resolution.

Actual spaceflight studies have consistently reported an increase in alpha power [8, 9]. More specifically, the alpha rhythm in the parieto-occipital and sensorimotor areas was increased, possibly related to the decreased gravitational input in space [8]. Even more recently, it was found that there is an alpha decrease of the cerebellum and the vestibular network in microgravity, as well as in the bilateral motor cortex [9]. These findings underlie the increased processing necessary for postural stabilization, and the increased demand to integrate incongruent vestibular information [9].

In addition, several space analogue studies have been conducted to infer on the impact of microgravity and spaceflight on electrocortical function by means of EEG. A recent dry immersion study reported decreases in alpha power and a modest increase in theta power [10]. On the other hand, parabolic flight studies have shown a decrease of beta power, which is suggested to reflect either emotional reactions to weightlessness [11], baroreceptor stimulation [12] or lower arousal levels [13]. Finally, HDBR studies have corroborated on the increase in alpha power as seen during actual spaceflight, but have also reported contrasting findings [14]. As stated above, the gravitational stimulus is preserved in HDBR, so it might not be the best model to investigate the effect of microgravity on the human brain [7].

In general, spaceflight-related alterations in electrocortical activity have been proposed to reflect mainly emotional stressors [7]. For example, parabolic and spaceflights are associated with increased stress levels (e.g., anxiety), while dry immersion and HDBR on the other hand are associated with boredom due to monotony and immobilization [7].

## Magnetic resonance imaging (MRI) studies

MRI is an imaging technique that allows measuring structural, functional, metabolic, and vascular effects in vivo. MRI allows to investigate, among others, brain structure, structural connectivity, and functional connectivity.

So far, only one study has reported MRI-based findings related to actual spaceflight. This one case study has shown a decreased intrinsic (functional) connectivity in a vestibular-related cortical area, i.e. the right insula [15], underlying the effect of gravitational and vestibular deprivation. Furthermore, a decrease in cerebellar-motor connectivity was found postflight [15]. This case study suggests that typical spaceflight-related problems have both a central and peripheral component, although generalization should be made very carefully. Ongoing longitudinal studies are aiming to extend these preliminary investigations in a larger cohort of astronauts.

Up until now, no MRI studies implementing dry immersion have been published. With regard to parabolic flight, there are no MRI-based data published yet. However, recently, a decreased intrinsic connectivity strength in the right angular gyrus (rAG) was observed [16], a region in the temporoparietal junction (TPJ), known to be involved in multisensory integration, as well as in cognitive and spatial tasks (e.g., [17]). This might resemble the downregulation of conflicting vestibular signals during microgravity. Finally, a multitude of MRI-based HDBR studies has been conducted, both of short and long durations. In general, the studies have primarily reported alterations related to motor-related tasks (e.g., fine motor control [18]) and more advanced cognitive function such as executive function [19] and spatial working memory [20]. Consequently, most studies found changes in resting-state functional connectivity, as well as white and grey matter in sensorimotor, somatosensory and cognitive-related brain regions (for a full overview, readers are referred to [21]). The scarce involvement of the vestibular-related structures in HDBR studies is probably related to the remaining gravitational input.

## General difficulties and future perspectives

Previous spaceflight and space analogue studies have already shown that there is a large individual variability for several physiological processes such as vestibular deconditioning and sensorimotor adaptation. In addition, neural adaptation is known to vary quite strongly and can be influenced by several factors such as demographics, genetics, and physical activity. Furthermore, EEG studies have reported microgravity effects on (electro)cortical activity to be task-dependent. This makes generalization difficult. Another typical spaceflight factor adding to this is the inherent small sample sizes in space research. Indeed, it is very difficult to acquire data in a large group of space travellers within a reasonable time frame. The extended periods, often years, during which data are collected, might, therefore, lead to changes in settings (e.g., acquisition parameters), equipment and team members, possibly confounding the observed changes.

In addition, from a logistic point of view, it is often impossible to assess space travellers in the first few hours/days after re-entry. However, especially in view of central adaptation processes, it is desirable to assess as soon as possible after re-exposure to Earth’s gravity. There might be a critical time frame to probe certain changes. A first assessment after a few days might, therefore, miss effects of interest and/or confound observed changes, as there is already a re-adaptation to Earth’s conditions [15]. Therefore, investigations should be done at well-considered and repeated timepoints to infer on the exact temporal profile and longevity of (electro)cortical changes.

Furthermore, certain assessment techniques such as MRI are restricted to ground-based measurements only, i.e., preflight and postflight measurements. Therefore, it is recommended to implement multimodal research protocols, complementing MRI assessments with, e.g., EEG, transcranial magnetic stimulation or near-infrared spectroscopy to get a better insight into the changes occurring at the level of the brain both during and after spaceflight. Concomitantly, the limitations inherent to a single technique are overcome. For example, combining EEG and MRI would improve spatio-temporal resolution.

## Conclusions

Currently, there is paucity in the knowledge of the effect of spaceflight and microgravity on the human brain, complicating advancement of space neurosciences. Overall, cerebellar, sensorimotor, and vestibular brain regions seem to be affected most. Previous vestibular-related space research has focused primarily on peripheral hampered function, but the current research suggests that the central deconditioning is at least as important. Elaborating on the understanding of how the brain reacts to and behaves in microgravity is a crucial step in the development of more adequate countermeasures against the detrimental changes often seen in space travellers early after return. This is pivotal to guarantee the safety and efficiency of future space missions. In addition, this acquired insight might eventually have relevant implications for patients suffering from neurodegenerative disorders, vestibular problems, and immobilization/inactivity.
